# Investigating the clinical significance of body composition changes in patients undergoing chemoradiation for oropharyngeal cancer using analytic morphomics

**DOI:** 10.1186/s40064-016-2076-x

**Published:** 2016-04-11

**Authors:** Chen Wang, Jeffrey M. Vainshtein, Maria Veksler, Patrick E. Rabban, June A. Sullivan, Stewart C. Wang, Avraham Eisbruch, Shruti Jolly

**Affiliations:** Department of Radiation Oncology, University of Michigan, UHB2C447, SPC 5010, 1500 East Medical Center Drive, Ann Arbor, MI 48109-5010 USA; Morphomic Analysis Group, University of Michigan, Ann Arbor, MI USA; Department of Radiation Oncology, Veterans Affairs Ann Arbor Health Care System, Ann Arbor, MI USA

## Abstract

**Background:**

The purpose is to investigate the clinical significance of body morphomics changes in stage III–IV oropharyngeal cancer patients during concurrent chemoradiotherapy (CRT).

**Methods:**

Fifty patients who underwent CRT were selected for body composition analyses by either availability of pre/post treatment DEXA scans or a novel CT-based approach of body morphomics analysis (BMA). BMA changes (lean psoas and total psoas area) were compared to total lean body mass changes by DEXA scans using two-sample *t* tests. Pearson correlation was used to compare the BMA measures to head and neck specific quality of life outcomes. Cox hazards model was used to predict mortality and tumor recurrence.

**Results:**

Clinically significant declines in total psoas area and lean body mass of similar magnitude were observed in both BMA and DEXA cohorts after CRT. Loss of psoas area (P < 0.05) was associated with greater frailty and mobility issues (3 out of 15 UWQOL domains). Total psoas area is more sensitive for local recurrence than weight changes and T-stage on multivariate analyses.

**Conclusions:**

BMA specifically evaluating psoas area appears to correlate with head and neck cancer quality of life physical domains. Pre- and post-treatment total psoas area at L4 appears prognostic for tumor recurrence.

## Background

Total body composition (TBC) is an analysis of patient muscle and fat distribution, and represents a global assessment of their state of health. Adverse TBC changes are widespread among cancer patients including those with breast cancer, lung cancer, gastrointestinal malignancies and gynecologic cancer (Awad et al. 2012; Fouladiun et al. 2005; Gil et al. 2006; Harvie et al. 2003; Jacquelin-Ravel and Pichard 2012; Silver et al. 2007; Tan et al. 2009; Smith 2004; Demark-wahnefried et al. 2001). Fluctuations in TBC during cancer treatment are patient-specific and generally vary according to disease site and treatment modality. Furthermore, TBC changes have been shown to be associated with impairments in physical function (Brown et al. 2005), quality of life, resistance to therapy and worse prognosis in some cancer patient populations (Fearon et al. 2011; Wallengren et al. 2013; Fearon et al. 2006; Couch et al. 2007; Tsai 2012). Given the potential clinical consequences of during and post-treatment TBC alterations, further understanding these changes and evaluating the potential need for intervention in response to TBC alterations is important.

Head and neck cancer patients, in particular, experience significant treatment and cancer-related weight loss due to a multifactorial metabolic syndrome (Silver et al. 2007). More than 70 % of this weight loss is attributed to loss of muscle mass (Silver et al. 2007), which occurs despite adequate nutritional supplementation. This loss of lean muscle mass without a corresponding loss in fat mass, known as sarcopenia, correlates with worse overall prognosis in oropharyngeal cancer patients (Adelstein et al. 2000). Better understanding of TBC in head and neck cancer patients is necessary to design interventions that may improve their quality of life, functional status, and overall prognosis. Moreover, there is need for a convenient and accurate technique to evaluate changes in body composition. Dual-energy X-ray absorptiometry (DEXA) has been the gold standard for determining total body composition; however, its accuracy may be limited in patients undergoing large changes in volume status, such as with head and neck cancer patients undergoing treatment (Jackson et al. 2013). Past studies characterizing changes in TBC have utilized different methods based on fundamentally different techniques, including plain anthropometric measurements (Hyltander et al. 1991; Richards et al. 2012), neutron activation analysis (MacFie and Burkinshaw 1987), bioelectric impedance measurements (Simons et al. 1995), computerized tomography (Smith 2004), and DEXA (Jackson et al. 2013; Maturo et al. 2003; Koch 1998). These methods may be difficult to obtain at the respective institution, and they have yielded inconsistent results due to intrinsic measurement limitations and variations (Fouladiun et al. 2005).

A recently developed novel technique utilizes a high throughput analysis of body composition characteristics (body morphomics analysis) to assess patient frailty. This innovative CT-based approach offers convenience for the patients and limits additional tests as most patients with cancer undergo routine imaging with CT before and after chemoradiation. Furthermore, body morphomics offers significant cost saving implications by reducing additional tests and supports patient-centered individualized cancer care planning.

Body morphomics analysis can rapidly and accurately characterize the changes in regional body composition in cancer patients, and has shown a strong correlation between trunk muscle (psoas) size and mortality in multiple clinical settings, including postoperatively following liver transplant surgery, abdominal aneurysm surgery, and inpatient general surgery (Zarinsefat et al. 2014; Levi et al. 2014; Waits et al. 2014; Englesbe et al. 2013; Sheetz et al. 2013). Core muscle size has similarly shown prognostic value in patients with esophageal cancer (Sheetz et al. 2013), adrenocortical carcinoma (Miller et al. 2012), and melanoma (Sabel et al. 2011). The role of body morphomics in head and neck cancer patients treated with chemoradiotherapy (CRT), who experience significant metabolic disturbances during treatment, remains unexplored to date. Furthermore, to our knowledge no study has looked at the correlation between regional changes in psoas area as determined by CT with changes in total body composition as determined by DEXA scan, the current gold standard for body composition analysis. This is important to help us understand whether analysis of a single slice CT is sufficient for predicting changes in total body composition.

In this study of locally advanced head and neck cancer patients undergoing concurrent chemoradiation, we aim to characterize the changes in body morphomics (total psoas area, lean psoas area, psoas muscle density, HU) before and after CRT by determining the association between these changes with patient-reported quality of life (QoL) and tumor related outcomes. We hypothesize that decline in psoas area as determined by CT at L4 vertebral body level is associated with worse quality of life and tumor related outcomes. Our second aim was to determine whether changes in psoas area correlate with changes in total body composition as determined by DEXA scan. We hypothesize that psoas area will decline similarly to TBC changes of lean body mass by DEXA scan during CRT.

## Methods

After obtaining University of Michigan Institutional Review Board approval, 93 patients were enrolled in 2 consecutive prospective trials of organ sparing chemotherapy with intensity-modulated radiation therapy (IMRT) for locally advanced oropharyngeal cancer from 2003 to 2011. Eligibility included stages III to IV squamous cell carcinoma of the oropharynx, no prior therapy, Karnofsky performance status ≥60, and primary therapy with CRT. Details of therapy have been previously published (Hunter et al. 2013; Feng et al. 2010). Briefly, all patients required treatment of bilateral neck. All patients received concurrent weekly chemotherapy with carboplatin (area under curve = 1) and paclitaxel (30 mg/m^2^). None received induction or adjuvant chemotherapy. The IMRT prescription doses were 70 Gy to the primary tumor and involved lymph nodes, 59–63 Gy to high-risk nodal regions and the anatomic compartments around the gross tumor volumes, and 56 Gy to low-risk nodal regions, all delivered in 35 fractions. Priority was given during planning to spare the noninvolved parts of the major salivary glands, the oral cavity, pharyngeal constrictor muscles, larynx, and the esophagus, to minimize late xerostomia and dysphagia. Feeding tubes were inserted if weight loss during therapy approached 10 %. All procedures were in accordance with the ethical standards of the committee on human experimentation of the institution or in accord with the Helsinki Declaration of 1975 as revised in 1983. Details of the radiation treatment planning, the dosimetric and functional results in these patients have previously been published (Hunter et al. 2013; Feng et al. 2010).

In a separate study, after obtaining University of Michigan Institutional Review Board Approval, 12 patients with locally advanced squamous cell head and neck cancer were enrolled in a prospective study assessing the overall changes in total body composition using DEXA. This cohort is a subset of a larger cohort that is being accrued for a study assessing the impact of exercise training on changes in total body composition for patients receiving concurrent chemoradiation therapy (CCRT) for locally advanced head and neck cancer. Eligibility criteria included patients with American Joint Committee on Cancer stage II–IV squamous cell head and neck cancer receiving CCRT as first-line treatment without surgery. All patients underwent a preliminary nutritional assessment by a dietician before initiation of treatment. Six patients received a percutaneous endoscopoic gastrostomy (PEG) tube before initiation of radiation therapy, and an additional 3 patients received a PEG tube during radiation therapy for enteral nutrition. If a patient experienced >5 to 10 % decrease in body mass during the course of treatment, a nutritionist was consulted. All procedures were in accordance with the ethical standards of the committee on human experimentation of the institution or in accord with the Helsinki Declaration of 1975 as revised in 1983. Details of the study have been previously published (Jackson et al. 2013).

Forty-three of ninety-three patients with PET/CT scans obtained pre-treatment and within 3–6 months following completion of treatment, for whom the CT scan included the psoas muscle, were eligible for the present study, and other fifty patients were initially treated at outside institutions and their staging CT scans were not available for analysis. Twelve patients with DEXA-scan based body composition analyses obtained pre-CRT and 2 months post-CRT follow-up were also enrolled (Jackson et al. 2013). Clinical data including age, sex, height, weight, dosimetric data, smoking status, tumor recurrences, mortality, and cancer stage were obtained for the patients.

Body morphomics analysis (BMA) was performed on pre- and post-treatment chest and abdominal computed tomography (CT) in a semi-automated fashion using algorithms programmed in MATLAB v13.0. To determine the cross sectional areas and densities of the left and right psoas muscle at the level of the fourth lumbar vertebra (L4), individual vertebral levels were initially identified on each patient’s PET-CT scan in sagittal section. Individual transverse imaging slice at the superior aspect of L4 were selected and the borders of the left and right psoas muscle were outlined using an edge-detecting algorithm, and verified by trained investigators. The areas of the resulting enclosed regions were then computed and summed to yield the total cross-sectional area of the psoas muscle (Englesbe et al. 2013). Average density in Houndsfield Units (HUs) of the psoas muscle in the selected regions was also measured. To adjust for fatty infiltration of the psoas muscle, the average density was used to calculate the total cross-sectional area of the psoas muscles, excluding fatty infiltration (lean psoas muscle) (Englesbe et al. 2012).

Head-and-neck cancer and treatment-related QoL was assessed by 2 validated instruments: the Head and Neck QOL (HNQOL) (Koch 1998) and University of Washington QOL (UWQOL) (Hassan and Weymuller 1993), version 3. Briefly, UWQOL (version 3) is a validated, self-administered, and multi-factorial, questionnaire that provides questions specific to head and neck cancer. Ten questions domains of UWQOL included: pain, appearance, activity, recreation, employment, chewing, speech, taste, saliva, and mucous. Scoring is scaled so that a score of 50 represents the worst possible response, and a score of 10 represents the best possible response. Scoring is scaled in equal increments from ten to fifty to reflect the number of possible responses. Thus the pain domain has 5 possible responses, which are scored as 10, 20, 30, 40, and 50.

The Head and Neck QOL is a validated and self-administered questionnaire also specific to head and neck cancer. This questionnaire had four domains: eating (6 items), communication (4 items), pain (4 items), and emotion (6 items). Scoring is scaled so that a score of 4 represents the worst possible response, and a score of 0 represents the best possible response. Scoring is scaled in equal increments from 0 to 4 to reflect the five possible responses. For example, within the communication domain, “ability to talk to other people” has 5 possible responses, which are scored as 0, 1, 2, 3, and 4. HNQOL and UWQOL questionnaires were administered at 6 time intervals: before initiation of treatment and at 3, 6, 12, 18, and 24 months after treatment.

DEXA measurements of total lean body mass and body morphomics parameters of total psoas area and lean psoas area were the main variables of interest from the respective analysis. Normal distribution of the fore mentioned variables was verified with Q–Q plots. BMI categories of normal (18.5–24.9), overweight (25–29.9), and obese (≥30.0) were used to stratify for further analysis of weight loss. Changes in weight, BMI, total psoas area, lean psoas area, and lean body mass were characterized with mean, standard deviation, percent change, two-sample *t* test, and 95 % confidence interval. Two-sample *t* test was employed to compare the percent change of lean body mass with that of total and lean psoas area. Cox proportional hazards model as used to assess the effect of total psoas area on tumor recurrence and mortality. The Pearson’s correlation coefficient was calculated to evaluate associations between changes in BMA measures from pre-treatment to 3 months post-treatment with changes in UWQOL and HNQOL from pre-treatment to 6 months post-treatment. We chose to analyze the quality of life associations at the fore mentioned time frame because patients were still recovering from CRT side effects at 3 and at 12–24 months, deterioration in quality of life began to return to pre-treatment levels. A significance level of *α* = 0.05 was used. All statistical analysis was performed using SPSS (IBM Corp. Released 2013. IBM SPSS Statistics for Windows, Version 22.0. Armonk, NY: IBM Corp.).

### Consent

Written informed consent was obtained from the patients to participate in this study and for publication of this research article and any accompanying images.

## Results

Forty-three of the 93 patients enrolled in prospective phase II chemo-IMRT had pre-treatment and post-treatment follow-up PET/CT scans available for morphomics analysis and thirty-eight of the forty-three had also prospectively completed the UWQOL and HNQOL questionnaires. Twelve other patients had pre- and post-treatment DEXA scans available for total body composition analysis. Characteristics of the two groups are summarized in Table 1. DEXA study patient group and BMA group are similar with respect to age, sex, tumor site, AJCC stage, and IMRT dose. Clinical and demographic similarities between the two groups allow for comparison between total body composition changes and total psoas muscle area changes.

**Table 1:** Clinical characteristics and total body composition changes

	DEXA study	Body morphomics analysis
Sample size	N = 12	N = 43
Age (years)	57 (±8.1)	57 (±7)
Weight (kg)	93 (±12.6)	92 (±17.4)
BMI	27.1 (±3.0)	29.1 (±5)
LBM (kg)	59.2 (±7.8)	–
FBM (kg)	30.6 (±7.5)	–
Total psoas area (mm^2^)	–	2811 (±726)
Lean psoas area (mm^2^)	–	1407 (±356)
Male	12 (100.0)	36 (95.0)
Female	0 (0.0)	2 (5.0)
Tumor site		
Base of tongue	6 (50.0)	20 (53.0)
Tonsil	4 (33.3)	18 (47.0)
Other	2 (16.7)	–
AJCC stage		
III	7 %	5 %
IVA	83 %	95 %
IVB	–	–
Treatment modality, CCRT	12 (100.0)	38 (100.0)
IMRT dose, Gy	70.0 (±0.0)	70.0 (±0.0)

*LBM* lean body mass, *FBM* fat body mass, *CCRT* concurrent chemo-radiation therapy, *Gy* gray dosage

Clinically significant lean muscle mass decline was seen in both BMA and DEXA cohorts, shown in Table 2. Decrease in overall lean body mass by DEXA scan was 10.2 %, which is comparable to the degree of decline in total psoas area by body morphomics of 10.9 %. Lean psoas area on the other hand seemed to over-estimate the degree of lean body mass loss; it decreased by 14.1 % after CRT. Psoas Houndsfield unit (HU), a measure of muscle density, decreased by 9.2 %, which is indicative of increase in fatty infiltration and decline in muscle quality after CRT. Decreases in weight were similar between the DEXA and BMA group (11.7 and 10.33 %, respectively). Summary of body morphomics changes are shown in Table 3.

**Table 2:** DEXA total body composition changes

	Net change	Percent change	P value
BMI	1.75 (±2.2)	5.96	<0.05
Weight (kg)	11.2 (±6.9)	11.70	<0.05
Lean body mass (kg)	6.1 (±6.8)	10.20	0.001

In the DEXA scan cohort, changes in BMI, weight and lean body mass were assessed at pre- and 2 months post-treatment follow-up

**Table 3:** Body morphomics changes

Characteristics (N = 43)	Pre-treatment Mean (SD)	Post-treatment Mean (SD)	Percent change (95 % CI)	P value
Weight (kg)	92 ±17	82 ± 15	−10.33 (−8.05, −12.61)	<0.001
Body mass index (BMI)*	29.1 ± 5	26.7 ± 4.2	−9.71 (−7.41, −12.01)	<0.001
Normal (N = 8)	23.2 ± 1.14	21.2 ± 2.89	−8.62 (−5.6, −11.64)	<0.05
Overweight (N = 16)	28.20 ± 1.41	25.72 ± 2.03	−8.79 (−7.39, −10.19)	<0.001
Obese (N = 19)	34.71 ± 3.18	29.86 ± 3.03	−13.97 (−11.7, −16.24)	<0.001
Bone Marrow Density (L4)	159 ± 42.1	159 ± 41.3	0	–
Total psoas area (mm^2^)	2811 ± 726	2492 ± 626	−10.88 (−8.70, −13.06)	<0.001
Left psoas area (mm^2^)	1407 ± 356	1244 ± 311	−10.75 (−7.73, −13.77)	<0.001
Right psoas area (mm^2^)	1404 ± 385	1248 ± 328	−10.74 (−8.45, −13.03)	<0.001
Psoas density (HU)	52.0 ± 7.1	47.2 ± 7.9	−9.16 (−4.42, −13.9)	<0.005
Lean psoas (HU × mm^2^)	1130 ± 300	973 ± 270	−14.09 (−11.07, −17.01)	<0.001

Changes in body morphomics measures were assessed from pre-treatment to 3 months post-up

* BMI categories of normal (18.5–24.9), overweight (25–29.9) and obese (≥30) were used to stratify the cohort

In our BMA cohort, there were nine patients with tumor recurrences and six deaths. In univariate analysis, greater percent decline in total psoas area was correlated with higher risk of tumor recurrence [hazard ratio (HR) = 1.117; 95 % CI 1.018, 1.225; P = 0.019] and mortality (HR = 1.127; 95 % CI 1.018, 1.225; P = 0.019), Table 4. The interpretation is for each 1 % loss of total psoas area, the risk of tumor recurrence and mortality increases by 11.7 and 12.7 % respectively. Given that our patients on average loose 10.9 % of total psoas area, the risk of tumor recurrence and mortality is 127.3 and 138.2 %, respectively, higher relative to a patient without any changes in total psoas area.

**Table 4:** Univariate analysis of risk factors and adverse patient outcomes

Measure	P value	HR (95 % CI)
(A) Tumor recurrence		
Total psoas area (% decline)	0.019	1.117 (1.018–1.225)
Weight (% decline)	0.163	1.064 (0.975–1.160)
Age	0.43	0.961 (0.871–1.061)
T-stage	0.043	2.048 (1.024–4.095)
Lean psoas area (% decline)	0.14	1.047 (0.985–1.114)
Psoas HU (% decline)	0.933	0.988 (0.957–1.041)
(B) Mortality		
Total psoas area (% decline)	0.043	1.127 (1.004–1.266)
Weight (% decline)	0.047	1.111 (1.001–1.233)
Age	0.0514	1.037 (0.929–1.157)
T-stage	0.028	3.891 (1.161–13.046)
Lean psoas area (% decline)	0.147	1.060 (0.980–1.148)
Psoas HU (% decline)	0.656	1.010 (0.966–1.057)

Higher percent decrease in weight in univariate analyses was correlated with higher risk of mortality (HR = 1.111; 95 % CI 1.001, 1.233; P = 0.047) but not tumor recurrence (P = 0.163). As expected, T-stage also correlated with tumor recurrence (HR = 2.048; 95 % CI 1.024–4.095; P = 0.043) and mortality (HR = 3.891; 95 % CI 1.161–13.046; P = 0.028) in univariate analyses. The interpretation is for each increase in stage (i.e. from stage III to IV), the risk of tumor recurrence and mortality increases by 104.8 and 289.1 % respectively. Lean psoas area and psoas density (HU) did not correlate with tumor recurrence or mortality in univariate analysis.

In multivariate analysis adjusted for patient age, percent decrease in weight and tumor stage, greater decline in total psoas area was a significant predictor of tumor recurrence (HR = 1.148; 95 % CI 1.005, 1.311; P = 0.042), but not mortality (P = 0.173), Table 5. While tumor stage was a significant predictor in multivariate analysis of tumor recurrence, when adjusting for total psoas area as well as patient age, and weight, T-stage was not significant in predicting mortality among patients (HR = 3.054; 95 % CI 0.960, 9.717; P = 0.094). On multivariate analyses adjusted for age, percent decrease in total psoas area, and T-stage, percent decline in weight was not a significant predictor of either tumor recurrence (P = 0.502) or mortality (P = 0.787). Of note, gender was not used as a covariate in multivariate analyses due to small number of females in the cohort (n = 2).

**Table 5:** Mulitivariate analysis of risk factors and adverse patient outcomes

Factor (% decrease)	Tumor Recurrence			Mortality		
	Hazard ratio (95 % CI)	P	AIC	Hazard ratio (95 % CI)	P	AIC
Total psoas area	1.148 (1.005–1.311)	0.042	44.104	1.111 (0.955–1.292)	0.173	40.657
Weight	0.966 (0.875–1.068)	0.502	41.496	1.016 (0.908–1.136)	0.787	40.341
Age	0.909 (0.805–1.026)	0.909	41.723	1.037 (0.891–1.207)	0.643	40.308
T-stage	2.305 (1.059–5.018)	0.035	43.682	2.878 (0.836–9.914)	0.094	41.387

AIC scores were calculated to determine the relative contribution of each covariate to model strength. AIC scores in Table 5 reflect model strength when the given covariate is removed from the model; higher AIC indicates greater importance of the removed covariate. Factors with P value <0.05 in the multivariate model are significant predictors of tumor recurrence or mortality. To shed light on the relative contribution of a factor, the AIC was calculated by the removal of that factor from the final model. Higher values of AIC indicates greater importance of the omitted factor. Total psoas area is a strong predictor, slightly more contributory than T-stage, of tumor recurrence (AIC-Psoas = 44.104; AIC-stage = 43.682).

Decrease in psoas muscle area from pre-treatment to first follow-up was significantly correlated with deterioration in 3 out of 15 UWQOL domains (activity, recreation/entertainment, and swallowing, all P < 0.05) as well as a single item in HNQOL regarding concerns for cancer related physical problems (P < 0.05) from pre- to 6 months post-treatment, Table 6. As expected, decline in psoas area are correlated with quality of life domains relating to mobility and maintenance of muscle function (i.e. swallowing). Body weight and BMI are correlated with 4 out of 14 UWQOL domains (general pain, recreation/entertainment, speech, and taste, all P < 0.05) and 3 out of 14 UWQOL domains (general pain, recreation/entertainment, and speech, all P < 0.05) respectively from pre- to 6 months post-treatment. No significant correlations were found between body weight and BMI with HNQOL. Summary of the correlations of changes in body morphomics with quality of life are shown in Table 6.

**Table 6:** Summary of body morphomics and quality of life correlations

	Body weight		Body mass index		Psoas muscle	
	Pearson’s r	P value	Pearson’s r	P value	Pearson’s r	P value
*UW QOL*						
Activity	−0.219	0.213	−0.189	0.284	−0.399	0.019
Chewing	−0.324	0.062	−0.289	0.097	−0.284	0.104
Disfigurement	−0.033	0.853	0.077	0.665	0.197	0.264
Employment	−0.056	0.753	−0.0004	0.998	0.033	0.853
General pain	−0.404	0.018	−0.374	0.029	−0.226	0.198
Mouth pain	−0.21	0.233	−0.197	0.264	−0.137	0.44
Mucus amount	−0.132	0.456	−0.119	0.503	−0.239	0.173
Mucus consistency	0.091	0.609	−0.08	0.653	0.014	0.937
Recreation/entertainment	−0.423	0.013	−0.378	0.028	−0.438	0.0096
Saliva amount	−0.031	0.862	0.0001	0.999	−0.048	0.787
Saliva consistency	0.159	0.369	0.217	0.218	0.157	0.375
Speech	−0.398	0.0197	−0.37	0.031	−0.252	0.151
Swallowing	−0.240	0.172	−0.226	0.199	−0.401	0.019
Taste	−0.338	0.05	−0.306	0.078	−0.066	0.711
Throat Pain	−0.194	0.272	−0.2	0.256	−0.152	0.291
*UM HNQOL*						
Emotion domain: concerns regarding…						
Cancer related physical problems	−0.143	0.419	−0.129	0.467	−0.453	0.007

Correlations between changes in body morphomics measures from pre-treatment to 3 months post-treatment with UWQOL and HNQOL measures from pre- to 6 months post-treatment. Of note, “concerns regarding cancer related physical problems” was the only measure that correlated with BMA out of the 4 HNQOL domains

Statistically significant associations between weight, BMI, and body morphomic parameter with quality of life domains are underlined

It is notable that tumor stage did not yield significant correlations with either HNQOL or UWQOL from pre- to 6 months post-treatment. Furthermore, common toxicity criteria (Trotti et al. 2000) also did not yield significant correlations with HNQOL or UWQOL from pre to 6 months post-treatment.

## Discussion

This is the first study to analyze the changes in body composition through a novel analytic morphomics technique utilizing single slice PET/CT at L4 in oropharyngeal cancer patients undergoing chemoradiation. We investigated the potential for using total psoas area by body morphomics to measure TBC changes. The study found similar declines in total lean body mass by DEXA, the current gold standard for TBC measurements (Jackson et al. 2013), and total psoas area by body morphomics, indicating that perhaps single slice CT total psoas area at L4 may adequately capture TBC lean body mass changes. Furthermore, body morphomics is performed on routine staging CT scans, offering patients convenience and limits the need for additional testing.

This study found significant declines in total psoas area, lean psoas area, and psoas HU from pre-treatment to 3 months post-treatment follow-up. Psoas muscle area decline is clinically significant because it correlates with deterioration in patient reported physical activity (3 out of 15 UWQOL domains) and concerns regarding cancer related physical problems (1 out of 4 HNQOL domains) from pre- to 6 months post-treatment on univariate analysis. Furthermore, decline in total psoas area independently predicts tumor recurrence in univariate and multivariate analysis adjusted for age, tumor stage, and decline in weight. In multivariate analysis, psoas muscle area loss also appears to be a greater contributor to tumor recurrence than T-stage, similar to results reported by Sheetz et al. (2013) who found lean psoas area to be a greater contributor than stage.

We found that lean psoas area and psoas density (HU) did not correlate with tumor recurrence or mortality in univariate analysis. This finding is in contrast to results reported by Sheetz et al. who conducted a similar study with esophageal cancer patients undergoing esophagectomy. They found lean psoas area to be predictive of overall survival and disease free survival on univariate analysis and multivariate analysis (Sheetz et al. 2013). This discrepancy shows that the sensitivities of different body morphomics measures (total psoas area vs. lean psoas area) in different clinical settings are not fully understood and require further research.

This study also found significant changes in body weight from pre-CRT baseline to post-CRT, a degree of loss that is comparable to findings from similar studies (Jackson et al. 2013). From our study, the effects of weight loss appear to hold less clinical significance than decline in total psoas area. Decline in body weight did not predict tumor recurrence in univariate or multivariate analysis. On univariate analysis, weight loss is associated with greater risk of mortality; however, this relationship is not significant on multivariate analysis adjusted for age, percent decline in total psoas area, and T-stage. In the multivariate models, psoas area also appears to be a stronger predictor for tumor recurrence than weight loss. Weight loss correlated with deterioration in general quality of life (i.e. general pain, recreation and entertainment, taste, and speech) domains from pre- to 6 months post-treatment on univariate analysis. This is in contrast with quality of life deterioration associated with total psoas area, which are specific to frailty and mobility.

There is a need for measurements of BMI to better quantify the interpersonal variations in the significance of weight loss (Fearon et al. 2006) and few studies have examined the changes in BMI in cancer patients in general. We examined the changes in BMI in oropharyngeal cancer patients and found that on average BMI decreased by 9.7 %. By stratifying patients into BMI categories of obese, overweight, and normal, we found distinct patterns of weight losses specific to each group. Decrease in BMI correlated with declines in 3 out of 15 UWQOL domains from pre- to 6 months post-treatment on univariate analysis. There is significant overlap in the associated QOL domains between BMI and weight loss. This indicates that future studies may only need to focus on one or the other when characterizing quality of life outcomes.

Our finding that total psoas area predicted mortality in oropharyngeal cancer patients on univariate analysis is not surprising given total psoas area decline is associated with increased mortality in multiple clinical settings (Zarinsefat et al. 2014; Levi et al. 2014; Waits et al. 2014; Englesbe et al. 2013; Sheetz et al. 2013; Miller et al. 2012; Sabel et al. 2011). It is surprising that decline in psoas area independently predicted tumor recurrence adjusted for age, T-stage, and weight loss. This is the first study to our knowledge to delineate this relationship. It is possible that patients who are more likely to experience tumor recurrence may have declines in body composition that portends the recurrence and can be readily detected by body morphomics; thus, in that subset of patients, body composition changes is reflectively of ongoing metabolic and inflammatory derangements of the patients.

Despite these findings, there are several limitations in this study. The study cohort represents a subset of previously published patients (Feng et al. 2010); our cohort size was limited by inclusion criteria of having pre- and post-treatment PET/CT as well as prospective completion of UWQOL and HNQOL questionnaires. Furthermore, our sample size (n = 38) limits the possibility of multivariate analysis for quality of life correlations. Future studies with larger patient cohorts are required to better delineate the relationship between decline in total psoas and deterioration in quality of life. Such studies may allow clinicians to prospectively identify patients who should undergo exercise and nutritional interventions to improve quality of life into survivorship (Jackson et al. 2013).

In conclusion, to better aid in the understanding of changes in detailed body composition, body morphomics analysis is an accurate and convenient method for patients to characterize body composition changes in cancer patients using the patient’s staging CT (or PET/CT) scans. Notably, body morphomics appears to be a more sensitive measure of adverse outcomes (i.e. tumor recurrences) than tumor stage and weight loss. Body morphomics is also associated with clinically significant changes in QOL up to 6 months post-CRT.

Given the accessibility of data in already existing staging and follow-up CT scans of head and neck cancer patients and ability to accurately provide body composition data using body morphomics analysis, this methodology may prove to be a vital instrument in the longitudinal evaluation of cancer patients. It could help clinicians identify patients who are at increased risk for cachexia as well as monitor the effects of behavioral interventions (diet and exercise) in cancer patients. More research in body morphomics in cancer patients is necessary to better understand its broad clinical application.
